# Nutritional Properties of Larval Epidermis and Meat of the Edible Insect *Clanis bilineata tsingtauica* (Lepidoptera: Sphingidae)

**DOI:** 10.3390/foods10122895

**Published:** 2021-11-23

**Authors:** Ying Su, Ming-Xing Lu, Li-Quan Jing, Lei Qian, Ming Zhao, Yu-Zhou Du, Huai-Jian Liao

**Affiliations:** 1 College of Horticulture and Plant Protection & Institute of Applied Entomology, Yangzhou University, Yangzhou 225009, China; wulisuying@163.com (Y.S.); lumx@yzu.edu.cn (M.-X.L.); zhaoming@yzu.edu.cn (M.Z.); 2 Jiangsu Key Laboratory of Crop Genetics and Physiology/Co-Innovation Center for Modern Production Technology of Grain Crops, Yangzhou University, Yangzhou 225009, China; lqjing@yzu.edu.cn; 3 Institute of Leisure Agriculture, Jiangsu Academy of Agricultural Sciences, Nanjing 210014, China; qianlei14930@sina.cn; 4 Joint International Research Laboratory of Agriculture and Agri-Product Safety, Yangzhou University, Yangzhou 225009, China

**Keywords:** *Clanis bilineata tsingtauica* Mell, edible insects, nutritional composition, phytic acid

## Abstract

Insects represent a sustainable, protein-rich food source widely consumed in Asia, Africa, and South America. Eating *Clanis bilineata tsingtauica* Mell is common in the eastern part of China. A comparative characterization of nutrients in the meat and epidermis of *C. bilineata tsingtauica* was performed in this study. The results showed this insect to be high in nutrients, particularly in the epidermis where protein total was 71.82%. Sixteen different amino acids were quantified in *C. bilineata* *tsingtauica*, and the ratio of essential to nonessential amino acids in the epidermis and meat was 68.14% and 59.27%, respectively. The amino acid composition of *C. bilineata tsingtauica* is balanced, representing a high-quality protein source. Eight minerals were quantified in *C. bilineata tsingtauica*, including four macro and four trace elements. Fe in the epidermis and Zn in the meat were abundant at 163.82 and 299.31 μg/g DW, respectively. The presence of phytic acid impacted the absorption of mineral elements in food. We also detected phytic acid in *C. bilineata tsingtauica*. The molar ratio of phytic acid to zinc (PA/Zn) in *C. bilineata tsingtauica* was very low (3.28) compared to *Glycine max* and *Cryptotympana atrata*, which indicated that mineral utilization was high. In conclusion, this study confirms that *C. bilineata tsingtauica* is a highly nutritious food source for human consumption, and the results provide a basis for further consumption and industrialization of this edible insect.

## 1. Introduction

As the world population continues to increase, food shortage has become a serious global problem [1]. The possibility of using edible insects as food and feed was suggested in 1975 as a route to easing global food shortages [2,3] and has also been addressed by the Food and Agricultural Organization (FAO) [4,5]. Although insects are the largest and most diverse group of organisms on Earth, their implementation as a food source has not been fully utilized [6,7]. Over one million species of named insects are estimated to exist worldwide, and approximately 1900–2000 species are edible [8,9,10]. Humans primarily consume arthropods from one of the following five orders: Isoptera, Orthoptera, Coleoptera, Hymenoptera, and Lepidoptera [11,12,13]. Numerous studies have shown that these insects are of high nutritional value and contain considerable amounts of protein (20% to 76% of dry matter), amino acids, vitamins, and minerals [10,14,15,16,17,18,19,20]. Nutritional values are highly variable, however, because of the wide range of edible species and the variation in developmental stages and feeding substrates [3,17,21,22,23].

*Clanis bilineata tsingtauica* Mell, 1922, (Insecta: Lepidoptera: Sphingidae) is an important pest of *Glycine max* (Linn.) Merr. The history of *C. bilineata tsingtauica* as a food source dates back to the Chinese Qing Dynasty about two hundred years ago [24,25,26]. The consumption of the fifth instar larvae of *C. bilineata tsingtauica* is common in many regions of China, especially in the Jiangsu, Shandong, and Henan provinces [27], and Lianyungang (Jiangsu province) is a major production center. The production of *C. bilineata tsingtauica* in *G. max* amounted to approximately 3 × 10^4^ t/year, with an output value of nearly RMB 4.5 billion. Since the annual demand for larvae is about 10 × 10^4^ t/year, production has failed to satisfy consumption needs [28]. Recently, farmers in Jiangsu, Shandong, and Henan have begun growing *G. max* as a *C. bilineata tsingtauica* food source to meet consumer demand.

Many consumers believe that *C. bilineata tsingtauica* is rich in nutrients, primarily because it feeds on nutrient-dense *G. max* leaves. Tian et al. reported that the crude protein content of whole larvae was 65.5%, essential amino acids accounted for 52.84% of total amino acids, and the content of unsaturated fatty acids was as high as 64.17% [29]. It is important to mention that different regions of China exhibit different ways of preparing and eating *C. bilineata tsingtauica*. For example, the processing method in Lianyungang involves removing the larval head, squeezing the body with a round wooden stick to retain the internal meat, discarding the external epidermis, and then processing the remaining body parts into a delicious soup. However, in Xuzhou (Jiangsu Province), the intact larvae with skin are chopped and fried for consumption, whereas the larvae are consumed after grilling in Shandong [27]. The variation in preparation methods indicates that a comprehensive analysis of *C. bilineata tsingtauica* is warranted.

In humans, the scarcity of mineral elements such as iron and zinc has been a difficult problem to solve [30,31]. The bioavailability of minerals in plants and plant products is inhibited by phytic acid [32,33,34], a derivative of inositol with six phosphate groups [35,36]. Phytic acid (PA) negatively impacts the absorption of zinc and iron in compound diets [37,38,39,40]; however, there are relatively few studies on the presence of PA in edible insects that feed on plants [41,42]. In this study, we focused on the preparation of *C. bilineata tsingtauica* using protocols typical in Lianyungang. Changes in protein and amino acid contents in the meat and epidermis of *C. bilineata tsingtauica* were determined, along with analysis of minerals and phytic acid. At the same time, compared with *G. max* and *Cryptotympana atrata*, (recorded in the Pharmacopoeia of the People’s Republic of China and the Taiwan Herbal Pharmacopoeia to have medicinal value), the results of this study provide a scientific basis and reference for the nutritional value, processing methods, and further industrialization of *C. bilineata tsingtauica*.

## 2. Materials and Methods

### 2.1. Collection and Preparation of Materials

The fifth instar larvae of *C. bilineata tsingtauica* reared on *G. max* leaves were obtained from the Dongming Yellow River Beach Ecological Agriculture Co. Ltd. in Heze, Shandong Province, China (35°14′ N, 115°26′ E). The internal meat of the larvae was removed with round wooden sticks to separate the inner material and organs from the epidermis (skin). The meat included all insect tissues except the epidermis. Larval parts were then frozen at −70 °C and transferred to a drying oven (Hangzhou Lantian Instrument Co., Ltd., Zhejiang, China) at 65 °C for 24 h. Body parts were then macerated with a stainless steel grinder (FW-100, Zhejiang, China) fitted with a 100-mesh sieve, and stored in brown, wide-mouth bottles until needed.

*G. max* leaves were collected from the same field as *C. bilineata tsingtauica*. A rice huller (OHYA-25, OHYA Corporation, Tokyo, Japan) was used to remove *G. max* hulls, and seeds were then pulverized into powder with a stainless steel grinder (FW-100, Zhejiang, China). For comparison, cicada, *Cryptotympana atrata*, was obtained from the Dongming Yellow River Beach Ecological Agriculture Co., Ltd. (Shandong, China), and the grinding and storage processes were identical to that of *C. bilineata tsingtauica*.

### 2.2. Determination of Protein Content

Crude protein was determined by the Kjeldahl method using an automated Kjeldahl System (Buchi, Flawil, Switzerland). The protein in each sample was disintegrated by catalytic heating, which released ammonia that subsequently reacted with sulfuric acid to produce ammonium sulfate [43]. Ammonia was freed, gasified by alkaline distillation, absorbed with boric acid, and further titrated with sulfuric acid [42]. Nitrogen content was calculated as described previously, and protein content was calculated by multiplying the result by a conversion factor of 6.25 [43,44].

The contents of albumin, globulin, glutelin, and prolamin were determined as described by Ju et al. [45]. In brief, the sample was first defatted with hexane and then subjected to protein extraction. The above protein was extracted with distilled water, 50 g/L NaCl, 0.02 mol/L NaOH, and 700 mL/L ethanol in sequence [46].

### 2.3. Determination of Amino Acid Content

AccQ·Tag high-performance liquid chromatography (HPLC) was used to measure amino acid content, and all HPLC analyses were performed on a Waters 2695 HPLC system (Waters Corporation, Milford, MA, USA). About 0.10 g of the powder sample was weighed into a 10 mL glass bottle; then 5.00 mL of 6 mol/L HCl was added and the mixture was well-shaken and sealed. After the bottle was placed in an oven at 110 °C for 24 h, the samples were run through filters for quantification and the volume was adjusted to 50 mL. The filtrate (2.00 mL) was transferred into a cuvette, and low-pressure evaporation was used to remove HCl in a vacuum freeze drier (Labconco Corporation, Kansas, MO, USA). The concentrate thus obtained was dissolved in 2.00 mL of pure water, and the solution was filtrated with a 0.45 μm membrane filter. Then, 10 μL of filtrate, 70 μL of AccQ·Fluor buffer, and 20 μL of derivatizing agent (Waters Corporation, Milford, MA, USA) were transferred successively into the derivatization tube, and the mixture was warmed at 55 °C for 10 min in an oven (Hangzhou Lantian Instrument Co., Ltd., Zhejiang, China). Finally, amino acid content was determined by high-performance liquid chromatography (Waters 2695 HPLC separation unit, 2487 UV detector, Waters Co., Milford, MA, USA, and Empower management system, X&Y Solutions, Inc., Boston, MA, USA). The AccQ·Tag reversed-phase analysis column was 3.9 mm × 150 mm; mobile phase A was sodium acetate (140 mmol/L)-ethylamine (17 mmol/L, pH 4.95, adjusted with phosphoric acid); mobile phase B was acetonitrile; and mobile phase C was pure water. Flow velocity was 1.00 mL/min; column temperature was 37 °C; UV detector wavelength was 248 nm; sample volume was 10 μL. The standards of 17 amino acid components were provided by Waters Co. Ltd. (Milford, MA, USA), except for tryptophan (Trp) [44].

A Waters 2695 HPLC detection system was used to determine the quantity of seven essential amino acids, including threonine (Thr), valine (Val), methionine (Met), isoleucine (Ile), leucine (Leu), phenylalanine (Phe), and lysine (Lys), but not tryptophan (Trp). The content of the following nine nonessential amino acids was also determined: aspartic acid (Asp), serine (Ser), glutamic acid (Glu), glycine (Gly), alanine (Ala), arginine (Arg), proline (Pro), histidine (His) tyrosine (Tyr), and cysteine (Cys) [44].

The following equations were used for evaluating amino acids:

$$\mathrm{Amino}\;\mathrm{acid}\;\mathrm{score}\;(\mathrm{AAS})=\frac{mg\;of\;amino\;acid\;in\;test\;protein}{mg\;of\;amino\;acid\;in\;FAO\;model}$$

$$\mathrm{Chemical}\;\mathrm{score}\;(\mathrm{CS})=\frac{mg\;of\;amino\;acid\;in\;test\;protein}{mg\;of\;amino\;acid\;in\;eggs}$$

$$\mathrm{Essential}\;\mathrm{amino}\;\mathrm{acid}\;\mathrm{index}\;(\mathrm{EAAI})=\sqrt[{n}]{\frac{Lys^{t}}{Lys^{s}}\times 100\times \frac{Trp^{t}}{Trp^{s}}\times 100\times \cdots \times \frac{Thr^{t}}{Thr^{s}}\times 100}$$

where *n* is the total number of essential amino acids, *t* is the amino acid content of the sample, and *s* is the amino acid content of the egg.

### 2.4. Determination of Mineral Content

Inductively coupled plasma–atomic emission spectroscopy (ICP-AES, ICAP 6300, Thermo, Waltham, MA, USA) was used for rapid and precise determinations of macro and trace mineral content in the samples. Briefly, pulverized samples (0.50 g) were weighed and combined with ultrapure-grade HNO_3_ (5.00 mL) and H_2_O_2_ (2.00 mL). A microwave digester (CEM MARS 5, Matthews, NC, USA) and the easy prep microwave digestion program were used to digest the samples [47]. After complete digestion, the mixed sample was cooled at room temperature and increased to a final volume of 20 mL with ultrapure water. Then, macro elements (Ca, K, Mg, and P) and trace elements (Cu, Fe, Mn, and Zn) were determined using ICP-AES. The optimal instrumental conditions were maintained at 15 L/min for the stable plasma gas flow rate. The auxiliary and the nebulizer gas flow rate were kept at 0.2 and 0.8 L/min, respectively. The sample flow rate was 1.5 mL/min, and the power was 1500 W.

### 2.5. Determination of Phytic Acid Content

Pulverized samples (0.25 g) were weighed and combined with a 0.7% HCl solution (5.00 mL). This mixture was incubated in a constant-temperature oscillator at 25 °C and 150 rpm for 1 h. After centrifugation at 4000 rpm for 15 min at 4 °C, supernatants were collected. Supernatants (0.60 mL) were combined with 2.40 mL of deionized water and 0.50 mL of FeCl_3_ (0.1%, *w*/*v*); the mixture was shaken and then centrifuged at 3400 rpm for 10 min. The absorbance of supernatants was measured with a spectrophotometer (UV-1601 UV-VIS Spectrophotometer, Shimadzu Corporation, Tokyo, Japan) at 500 nm, and the amount of phytic acid was calculated using a phytic acid standard curve [44].

### 2.6. Statistical Analyses

Results are shown as means ± standard deviation (SD). Analysis of *C. bilineata tsingtauica* was compared with *G. max* and *C. atrata*. Data were stored in Microsoft Excel (2013) and analyzed with SPSS v. 16.0 statistical software.

## 3. Results

### 3.1. Classification and Determination of Total Protein

The total protein content of *C. bilineata tsingtauica* was higher than *G. max*, and more abundant in the larval epidermis (71.82%) than in the meat (64.24%) (Table 1). Four proteins, including albumin, globulin, glutelin, and prolamin, were identified in *C. bilineata tsingtauica*, and had different concentrations in the larval meat and epidermis. For example, glutelin was the predominant protein (meat, 34.35%; epidermis, 34.23%) and globulin was the least abundant (meat, 5.13%; epidermis, 3.70%). Prolamin content in the *C. bilineata* *tsingtauica* epidermis (11.67%) was double that found in the meat (5.27%). Interestingly, prolamin levels in the *C. bilineata tsingtauica* epidermis and *C. atrata* (11.02%) were eight-fold greater than levels in *G. max* (1.28%) (Table 1).

### 3.2. Essential and Nonessential Amino Acid Content

Sixteen amino acids were analyzed in *C. bilineata tsingtauica*, including seven essential amino acids (EAA) and nine nonessential amino acids (NEAA). Trp was not measured (an alkaline hydrolysis method is needed), and Cys could not be determined due to analytical methods and not necessarily because it was absent. The total amino acid content (TAA) of the *C. bilineata tsingtauica* meat and epidermis was 455.62 and 710.07 mg/g DW, respectively. Both EAA and NEAA were higher in the larval epidermis than in the meat (Table 2). In the epidermis, the EAA and NEAA contents were 287.75 and 422.33 mg/g DW, respectively. Furthermore, the EAA/NEAA ratio (meat, 59.27%; epidermis, 68.14%) and EAA/TAA ratio (meat, 37.18%; epidermis, 40.51%) were higher in the epidermis than in the meat. Among the seven EAAs, Leu content was the highest in the *C. bilineata tsingtauica* meat (40.81 mg/g DW), Lys was highest in the epidermis (59.05 mg/g DW), and the Met content was the lowest of the four EAAs in both the meat and epidermis. Among the nine NEAAs, the Glu content was highest in the *C. bilineata tsingtauica* meat (78.40 mg/g DW) and epidermis (99.21 mg/g DW), and the Pro content was the lowest (meat, 3.60; epidermis, 3.69 mg/g DW). All 16 amino acids exhibited a higher content in the epidermis than in the *C. bilineata tsingtauica* meat. The TAA, EAA, and NEAA contents in the four food sources were ranked from high to low as follows: *G. max*, *C. bilineata tsingtauica* epidermis, *C. atrata*, and *C. bilineata tsingtauica* meat. The contents of Thr, Met, Ile, Leu, Lys, Glu, and Arg in the epidermis of *C. bilineata tsingtauica* were higher than in *G. max* and *C. atrata*; but the Phe content of *G. max* (107.26 mg/g DW) was over four-fold higher than that of the *C. bilineata tsingtauica* meat (24.81 mg/g DW), and more than twice that of the *C. bilineata tsingtauica* epidermis (40.64 mg/g DW) (Table 2).

**Figure 1:**
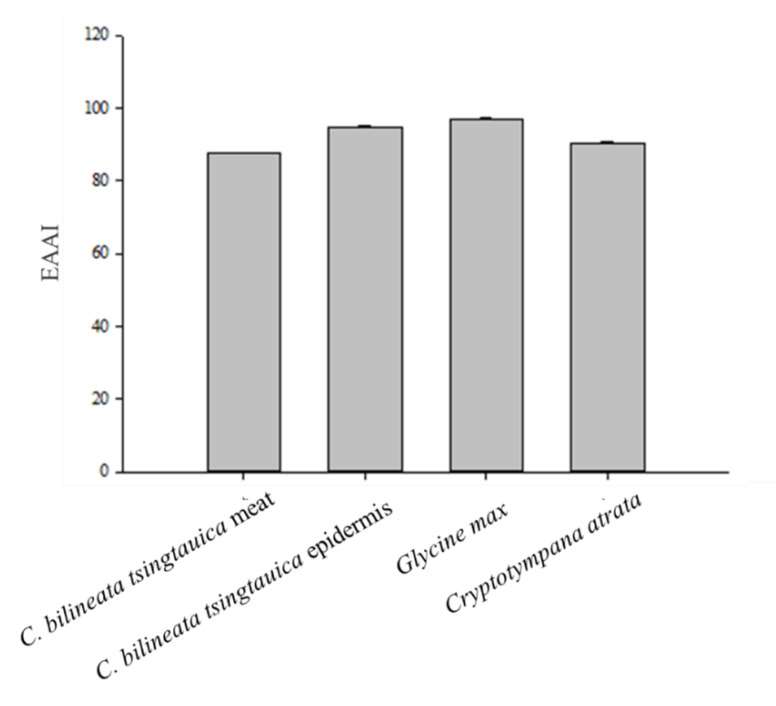
The essential amino acid index (EAAI) in *Clanis bilineata tsingtauica*, *Glycine max*, and *Cryptotympana atrata*.

The amino acid score (AAS), chemical score (CS), and essential amino acid index (EAAI) are important indicators to further evaluate the quality of amino acids. With respect to EAAs, our results showed the AAS of Met in *C. bilineata tsingtauica* was the lowest (meat, 0.27; epidermis, 0.54), which defined the first limiting amino acid (Table 3). The highest AAS score was that of the aromatic amino acids (Phe + Tyr) in the meat and epidermis of *C. bilineata tsingtauica*, where values were 0.72 (meat) and 1.33 (epidermis). The content of Ile, Lys, Phe + Tyr, and Thr in the epidermis of *C. bilineata tsingtauica* exceeded the amino acid content in the FAO standard, and the content of total essential amino acids was higher in the epidermis of *C. bilineata tsingtauica* compared to the FAO standard. Furthermore, the AAS and CS of essential amino acids in the epidermis of *C. bilineata tsingtauica* were higher than those in the meat (Table 3). *C. bilineata tsingtauica* possessed an excellent amino acid index of 87.67 in the meat and 94.91 in the epidermis (Figure 1). The overall EAAI was slightly lower for *C. bilineata tsingtauica* compared to *G. max*.

### 3.3. Determination of Mineral Content

The contents of four macro elements (calcium, potassium, magnesium, and phosphorus) and four trace elements (copper, iron, manganese, and zinc) were determined in *C. bilineata tsingtauica* (Table 4). Among the macro elements, Ca content was the highest in the meat (0.57 mg/g DW) and epidermis (0.73 mg/g DW), whereas Mg was the lowest (meat, 13.92; epidermis, 11.24 mg/g DW). As for trace elements in the *C. bilineata tsingtauica* meat, Cu content was the lowest (6.79 μg/g DW) and Zn was the highest (299.31 μg/g DW), whereas Mn was the lowest (11.04 μg/g DW) and Fe was the highest (163.82 μg/g DW) in the epidermis. The K, Mg, P, and Zn concentrations were higher in the *C. bilineata tsingtauica* meat than in the epidermis; however, the Ca, Cu, Fe, and Mn concentrations were higher in the epidermis than in the meat. Notable differences in the mineral content of *C. bilineata tsingtauica* and *G. max* included Fe in the epidermis and Zn content in the meat, which were about two- and six-fold higher than *G. max*, respectively (Table 4).

### 3.4. Phytic Acid (PA) Content and Mineral Bioavailability 

**Figure 2:**
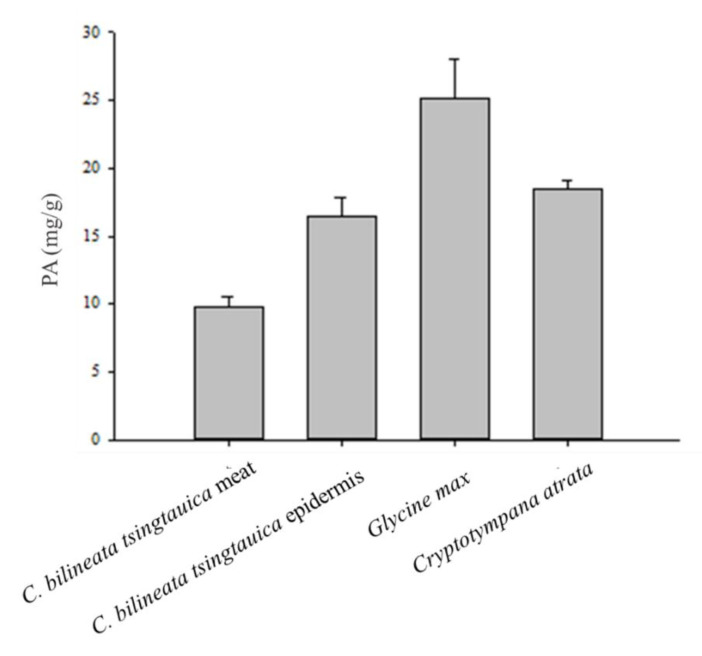
Phytic acid content in *Clanis bilineata tsingtauica*, *Glycine max*, and *Cryptotympana atrata* (DW).

**Figure 3:**
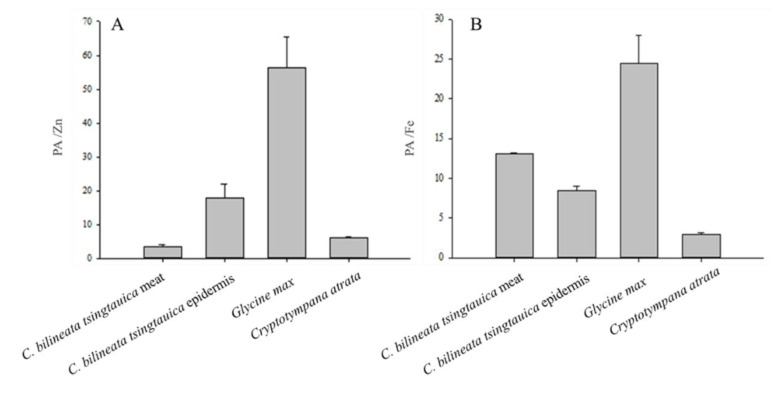
Bioavailability of Zn (**A**) and Fe (**B**) in *Clanis bilineata tsingtauica*, *Glycine max*, and *Cryptotympana atrata*.

Analysis showed that PA was present in both *C. bilineata tsingtauica* and *C. atrata*, but the concentration was lower than in *G. max* (Figure 2). The PA content of the *C. bilineata tsingtauica* epidermis (16.47 mg/g DW) was higher than that of the meat (Figure 2). The PA/Zn ratio in *C. bilineata tsingtauica* meat was 3.28, lower than the epidermis, *G. max*, and *C. atrata* (Figure 3). The PA/Fe ratio in *C. atrata* was 2.95, lower than *C. bilineata tsingtauica* and *G. max*. It is also important to note that the PA/Fe ratios in the *C. bilineata tsingtauica* meat and epidermis were lower than *G. max* at 13.13 and 8.52, respectively (Figure 3).

## 4. Discussion

According to the FAO, insects are an environmentally friendly food source for the growing world population [49]. As an edible insect, *C. bilineata tsingtauica* has a long history in China and is loved by the Chinese people. In this study, we found that *C. bilineata tsingtauica* is rich in protein, and the glutelin content is much higher than in *G. max*. Edible insects generally have a rich protein content and supply energy for various physiological functions [50,51,52,53,54]. Although many researchers have tried to substitute edible insect protein for meat protein [55], it is important to consider the risks of food allergy following insect ingestion [56], and possible pathogens in insects, too.

The essential amino acid composition of *C. bilineata tsingtauica* is relatively balanced and comprehensive. According to FAO standards, the ratios of EAA/NEAA and EAA/TAA in the *C. bilineata tsingtauica* epidermis meet the FAO/WHO recommended values (60% and 40%, respectively), while ratios in its meat are slightly lower [57]. Previous reports demonstrated that other edible insects, namely *Tenebrio molitor*, *Acheta domesticus*, and *Locusta migratoria*, contain seven essential amino acids, but the balance of EAAs is not as good as in *C. bilineata tsingtauica*. For example, the EAA/NEAA ratios in *T. molitor*, *A. domesticus*, and *L. migratoria* are only 57.32%, 55.04%, and 56.09%, respectively [58].

Minerals are essential micronutrients for animals and humans [59]. In this study, we measured the concentrations of eight minerals in *C. bilineata tsingtauica* and found that Zn is the highest in *C. bilineata tsingtauica* meat and Fe is highest in the epidermis. The recommended human intake of Fe and Zn is 15 mg/day [60,61], and Zn deficiency is highly prevalent in children and women in developing countries [62,63]. Additionally, phytic acid acts as an anti-nutrient by binding to Fe and Zn; this prevents the absorption of minerals in the gastrointestinal tract and decreases their bioavailability [64,65]. Phytic acid is present in numerous edible insect species (see Table 7 in [41]), and the PA/Zn and PA/Fe molar ratios of cereal and legumes are important considerations [66]. This study showed that the PA/Zn and PA/Fe ratios in *C. bilineata tsingtauica* are lower than in *G. max*, indicating that Zn, Fe, and bioavailability are higher in *C. bilineata tsingtauica* than in *G. max*. Therefore, the consumption of *C. bilineata tsingtauica* can alleviate the Fe and Zn deficiency caused by cereal- and bean-based diets in some areas [64,67], and can be used as a zinc supplement to reduce childhood morbidity and mortality in developing countries [68,69].

In conclusion, this study evaluated the nutritional value of *C. bilineata tsingtauica*, a rich source of protein and minerals. PA is present in *C. bilineata tsingtauica* and *C. atrata*, but the ratios showed that the bioavailability of minerals in these insects is superior to *G. max*. It is important to note that nutrition is distributed throughout the insect body, including the fat body in the abdomen and beneath the epidermis [70,71]. Based on our results, we advocate that the traditional way of eating *C. bilineata tsingtauica* in Lianyungang should be changed, and recommend the consumption of the entire larvae as is common in Xuzhou and Shandong. Future research should address the determination of toxic and beneficial substances in *C. bilineata tsingtauica*, and breeding technologies need to be improved to meet consumer demands for *C. bilineata tsingtauica* products.

## Figures and Tables

**Table 1:** The protein content (% dry weight) in *Clanis bilineata tsingtauica*, *Glycine max*, and *Cryptotympana atrata*.

	*Clanis bilineata tsingtauica* Meat	*Clanis bilineata tsingtauica* Epidermis	*Glycine max* (CK_1_)	*Cryptotympana atrata* (CK_2_)
Albumin	19.50 ± 2.40	22.25 ± 8.56	33.55 ± 2.05	12.75 ± 4.03
Globulin	5.27 ± 1.20	3.70 ± 0.57	4.56 ± 0.30	3.53 ± 0.39
Glutelin	34.35 ± 4.43	34.23 ± 3.49	8.17 ± 0.52	39.07 ± 3.20
Prolamin	5.13 ± 0.98	11.67 ± 1.32	1.28 ± 0.35	11.02 ± 2.01
Total protein	64.25 ± 0.21	71.85 ± 3.18	47.55 ± 1.63	66.35 ± 4.74

Note: Data are expressed as means ± SD (standard deviation).

**Table 2:** Amino acid content in *C. bilineata tsingtauica*, *Glycine max*, and *Cryptotympana atrata* (mg/g DW).

	*Clanis bilineata tsingtauica* Meat	*Clanis bilineata tsingtauica* Epidermis	*Glycine max* (CK_1_)	*Cryptotympana atrata* (CK_2_)
Thr *	23.35 ± 4.34	44.22 ± 2.81	42.37 ± 5.59	29.95 ± 1.51
Val *	22.85 ± 1.00	33.17 ± 4.12	37.85 ± 4.91	25.05 ± 1.78
Met *	9.49 ± 1.22	18.81 ± 1.21	10.40 ± 1.09	6.73 ± 0.74
Ile *	21.02 ± 1.71	40.79 ± 2.08	36.74 ± 4.41	29.73 ± 2.76
Leu *	40.81 ± 2.55	50.89 ± 6.54	41.23 ± 6.50	39.67 ± 2.67
Phe *	24.81 ± 3.10	40.64 ± 3.21	107.26 ± 6.93	34.65 ± 1.76
Lys *	26.91 ± 1.82	59.05 ± 2.38	48.73 ± 14.81	42.03 ± 1.51
Trp *	-	-	-	-
Asp	45.49 ± 7.47	66.59 ± 2.47	69.51 ± 7.28	64.38 ± 2.86
Ser	24.53 ± 4.26	35.12 ± 3.02	40.15 ± 1.53	31.56 ± 2.89
Glu	78.40 ± 2.18	99.21 ± 2.04	93.21 ± 4.42	97.59 ± 1.44
Gly	28.43 ± 1.84	42.37 ± 3.56	48.97 ± 2.99	30.74 ± 2.65
Ala	35.52 ± 3.28	47.76 ± 2.98	52.52 ± 4.02	28.34 ± 2.58
Tyr	18.38 ± 0.78	39.11 ± 3.04	58.96 ± 2.26	24.50 ± 2.69
Arg	30.92 ± 3.08	54.82 ± 4.94	40.30 ± 1.54	48.68 ± 0.52
Pro	3.60 ± 0.48	3.69 ± 0.11	6.28 ± 0.39	3.79 ± 0.45
His	21.13 ± 1.25	33.68 ± 2.24	39.60 ± 4.16	21.29 ± 3.14
Cys	-	-	-	-
EAA	169.23 ± 1.97	287.75 ± 15.49	324.56 ± 3.43	207.72 ± 7.87
NEAA	286.39 ± 20.95	422.33 ± 1.22	449.48 ± 16.70	350.84 ± 7.29
TAA	455.62 ± 22.92	710.07 ± 14.27	774.04 ± 20.13	558.55 ± 15.16
EAA/NEAA (%)	59.23 ± 3.64	68.14 ± 3.86	72.24 ± 1.92	59.20 ± 1.01
EAA/TAA (%)	37.18 ± 1.44	40.51 ± 1.37	41.94 ± 0.65	37.19 ± 0.40

Abbreviations: * essential amino acids (EAAs); -, not determined; EAA/NEAA, ratio of EAA and nonessential amino acids; EAA/TAA, ratio of EAA and total amino acids (TAA). Data are expressed as means ± SD.

**Table 3:** Comparison of the amino acid score and chemical score in *Clanis bilineata tsingtauica* with other sources.

	Content (mg/g DW)				AAS		CS	
	FAO *	Eggs *	Meat	Epidermis	Meat	Epidermis	Meat	Epidermis
Ile	40	52.4	21.02	40.99	0.53	1.02	0.40	0.78
Leu	70	84.1	40.81	50.89	0.58	0.73	0.49	0.61
Lys	55	64.9	26.91	59.05	0.49	1.07	0.41	0.91
Met	35	62.7	9.49	18.81	0.27	0.54	0.15	0.30
Phe + Tyr	60	95.5	43.19	79.75	0.72	1.33	0.45	0.84
Thr	40	53.9	23.35	44.22	0.58	1.11	0.43	0.82
Trp	10	16.2	-	-	-	-	-	-
Val	50	57.6	22.85	33.17	0.46	0.66	0.40	0.58
TAA	360	487.3	217.94	362.60	-	-	-	-

Note: * amino acid content as reported by Qiao et al. [48]; -, not determined. Abbreviations: AAS, amino acid score; CS, chemical score; FAO, Food and Agricultural Organization; TAA, total amino acids.

**Table 4:** Mineral element content in *Clanis bilineata tsingtauica*, *Glycine max*, and *Cryptotympana atrata* (DW).

	*Clanis bilineata tsingtauica* Meat	*Clanis bilineata tsingtauica* Epidermis	*Glycine max* (CK_1_)	*Cryptotympana atrata* (CK_2_)
Ca (mg/g) *	0.57 ± 0.12	0.73 ± 0.01	0.98 ± 0.03	1.59 ± 0.15
K (mg/g) *	12.53 ± 2.08	9.52 ± 0.13	13.56 ± 1.02	5.45 ± 0.16
Mg (mg/g) *	13.92 ± 2.21	11.24 ± 0.48	21.75 ± 0.49	12.09 ± 1.45
P (mg/g) *	7.91 ± 0.44	3.06 ± 0.15	5.10 ± 0.50	5.30 ± 0.24
Cu (μg/g)	6.79 ± 0.62	11.48 ± 0.29	14.00 ± 0.91	33.15 ± 0.16
Fe (μg/g)	63.05 ± 7.94	163.82 ± 4.08	87.88 ± 4.19	532.80 ± 30.92
Mn (μg/g)	10.57 ± 2.39	11.04 ± 0.02	23.37 ± 1.44	391.98 ± 13.51
Zn (μg/g)	299.31 ± 39.69	94.80 ± 20.57	44.71 ± 3.11	296.06 ± 33.54

Note: * macro element. Data are expressed as means ± SD.

## Data Availability

Not applicable.
